# Imaging findings for response evaluation of ductal carcinoma in situ in breast cancer patients treated with neoadjuvant systemic therapy: a systematic review and meta-analysis

**DOI:** 10.1007/s00330-023-09547-7

**Published:** 2023-04-05

**Authors:** Roxanne A. W. Ploumen, Cornelis M. de Mooij, Suzanne Gommers, Kristien B. M. I. Keymeulen, Marjolein L. Smidt, Thiemo J. A. van Nijnatten

**Affiliations:** 1 Department of Surgery, Maastricht University Medical Centre+, Maastricht, The Netherlands; 2 GROW – School for Oncology and Reproduction, Maastricht University, Maastricht, The Netherlands; 3 Department of Radiology and Nuclear Medicine, Maastricht University Medical Centre+, Maastricht, The Netherlands

**Keywords:** Breast neoplasms, Carcinoma intraductal noninfiltrating, Neoadjuvant therapy, Diagnostic imaging, Systematic review

## Abstract

**Objectives:**

In approximately 45% of invasive breast cancer (IBC) patients treated with neoadjuvant systemic therapy (NST), ductal carcinoma in situ (DCIS) is present. Recent studies suggest response of DCIS to NST. The aim of this systematic review and meta-analysis was to summarise and examine the current literature on imaging findings for different imaging modalities evaluating DCIS response to NST. More specifically, imaging findings of DCIS pre- and post-NST, and the effect of different pathological complete response (pCR) definitions, will be evaluated on mammography, breast MRI, and contrast-enhanced mammography (CEM).

**Methods:**

PubMed and Embase databases were searched for studies investigating NST response of IBC, including information on DCIS. Imaging findings and response evaluation of DCIS were assessed for mammography, breast MRI, and CEM. A meta-analysis was conducted per imaging modality to calculate pooled sensitivity and specificity for detecting residual disease between pCR definition no residual invasive disease (ypT0/is) and no residual invasive or in situ disease (ypT0).

**Results:**

Thirty-one studies were included. Calcifications on mammography are related to DCIS, but can persist despite complete response of DCIS. In 20 breast MRI studies, an average of 57% of residual DCIS showed enhancement. A meta-analysis of 17 breast MRI studies confirmed higher pooled sensitivity (0.86 versus 0.82) and lower pooled specificity (0.61 versus 0.68) for detection of residual disease when DCIS is considered pCR (ypT0/is). Three CEM studies suggest the potential benefit of simultaneous evaluation of calcifications and enhancement.

**Conclusions and Clinical Relevance:**

Calcifications on mammography can remain despite complete response of DCIS, and residual DCIS does not always show enhancement on breast MRI and CEM. Moreover, pCR definition effects diagnostic performance of breast MRI. Given the lack of evidence on imaging findings of response of the DCIS component to NST, further research is demanded.

**Key Points:**

• *Ductal carcinoma in situ has shown to be responsive to neoadjuvant systemic therapy, but imaging studies mainly focus on response of the invasive tumour.*

• *The 31 included studies demonstrate that after neoadjuvant systemic therapy, calcifications on mammography can remain despite complete response of DCIS and residual DCIS does not always show enhancement on MRI and contrast-enhanced mammography.*

• *The definition of pCR has impact on the diagnostic performance of MRI in detecting residual disease,* *and when DCIS is considered pCR, pooled sensitivity was slightly higher and pooled specificity slightly lower.*

**Supplementary Information:**

The online version contains supplementary material available at 10.1007/s00330-023-09547-7.

## Introduction

In recent decades, neoadjuvant systemic therapy (NST) has gained an increasing role in the treatment of both early-stage and locally advanced invasive breast cancer (IBC). The advantages of NST are in vivo evaluation of response to NST regimens and the decrease in tumour size, thereby increasing the likelihood of breast-conserving surgery and improving long-term outcomes [1–3]. Monitoring response to NST with the use of accurate imaging modalities is therefore important in surgical planning and estimation of prognosis [4]. Previous literature has indicated breast MRI is currently the most accurate imaging modality to monitor response of the primary tumour, yet a recent meta-analysis estimated similar accuracy of contrast-enhanced mammography (CEM) as well [5–9].

In approximately 45–60% of patients with IBC, a ductal carcinoma in situ (DCIS) component is present in the biopsy specimen at diagnosis [10–12]. DCIS has variable presentation, which hinders easy detection on imaging [13, 14]. On mammography, malignant calcifications or calcifications outside or adjacent to the mass can be considered suspicious for the presence of DCIS. However, 25% of DCIS cases do not contain mammographic calcifications [15, 16]. On breast MRI, DCIS tends to present as non-mass enhancement (NME); however, low-grade DCIS might lack enhancement [17, 18]. On CEM, DCIS can be detected based on the presence of enhancement and/or calcifications [19].

Many previous studies investigating response monitoring focused on predicting response of IBC rather than the presence of residual DCIS. Moreover, varying definitions for pathological complete response (pCR) are used in which residual DCIS is most often considered pCR [20]. On the contrary, accurate detection of residual DCIS is relevant, as it can be a cause for recurrence [21]. It was previously assumed that DCIS responds poorly to NST [22]. However, recent retrospective studies have demonstrated that DCIS adjacent to IBC can be fully eradicated after NST [12, 23, 24]. Consequently, the need to monitor the response of DCIS to NST by imaging, in addition to IBC response assessment, has increased in order to improve surgical planning.

Therefore, the aim of this systematic review and meta-analysis is to summarise and examine the current literature on imaging findings for different imaging modalities evaluating DCIS response to NST. More specifically, imaging findings of DCIS pre- and post-NST, and the effect of different pCR definitions, will be evaluated on mammography, breast MRI, and CEM.

## Materials and methods

### Literature search

This systematic review was performed according to the Preferred Reporting Items for Systematic Reviews and Meta-Analysis (PRISMA) statement [25]. PubMed and Embase databases were searched for eligible studies, and the last search was performed on August 9, 2022. Studies reporting mammography, breast MRI, and CEM results in predicting response to NST in the presence of IBC were included using the following keywords: breast neoplasm, ductal carcinoma in situ, mammography, contrast-enhanced mammography, magnetic resonance imaging, neoadjuvant systemic therapy, and other synonyms. References of included studies and relevant systematic reviews or meta-analyses were searched for additional eligible studies. There was no limitation for the year of publication, but only studies written in English were included. Supplemental Tables S1 and S2 show the full-search strategies used.

### Study selection

After removal of duplicates, titles and abstracts were screened and assessed for eligibility by two independent reviewers (R.P. and T.v.N.). Subsequently, full texts were read and considered eligible for inclusion if they met the predefined inclusion criteria: (1) mammography (MG), breast MRI, or CEM performed (before and) after completion of NST; (2) imaging findings correlated to postoperative pathology; (3) a clear description of the definition of pCR; and (4) information on the DCIS component related to imaging. It was decided to exclude conference abstracts, case reports and case series, animal studies, reviews, and articles on alternative treatment (e.g. neoadjuvant radiation therapy) or alternative imaging modalities (ultrasound, computed tomography). Regarding MRI methods, only dynamic contrast-enhanced (DCE) MRI data were used while diffusion-weighted imaging (DWI) was excluded. Any discrepancies during study selection were resolved in a consensus meeting between the reviewers.

Studies were eligible for meta-analysis when (1) the number of patients with pCR (ypT0) and residual DCIS without residual invasive tumour (ypTis) was reported and (2) post-NST data on true-positive (TP), true-negative (TN), false-positive (FP), and false-negative (FN) cases were provided per pCR definition (ypT0 versus ypT0/is). Studies were also included in the meta-analysis when the abovementioned information could be deduced from reported diagnostic performances.

### Data extraction and quality assessment

Data extraction was performed by two reviewers (R.P. and C.M.d.M.) independently, and any discrepancies were resolved by discussion with a third reviewer (T.v.N.). Collection of study information concerned study design, number and type of participants included, years of patient inclusion, and neoadjuvant treatment administered. For the imaging modalities used, information on vendor, settings, and imaging protocols was collected. Image evaluation during NST was summarised regarding subtraction images, region of interest analyses, computer-aided detection, and enhancement evaluation (subjective and/or objective, and specific late phase enhancement evaluation for DCIS detection [26, 27]). Definitions used for radiological complete response (rCR) and pCR in the included studies were summarised and evaluated. The definition of pCR was recorded as absence of residual invasive and in situ component (ypT0) or absence of residual invasive tumour, irrespective of the presence of residual DCIS (ypT0/is).

Quality of the included studies was assessed by the Quality Assessment of Diagnostic Accuracy Studies 2 (QUADAS-2) tool [28]. This tool comprises four domains: patient selection, index test, reference standard, and flow and timing. Domains were tested for risk of bias and concerns regarding applicability.

### Statistical analysis

For each imaging modality (mammography, breast MRI, and CEM), information on three topics was summarised: imaging findings of DCIS pre-NST, imaging findings of DCIS post-NST, and response evaluation of DCIS. Response evaluation consisted of studies investigating the response of DCIS according to imaging findings (i.e. pre- versus post-NST or correlation of imaging findings to potential response at histopathology of the surgical specimen).

The effect of pCR definition on diagnostic performance was investigated in a meta-analysis. Studies that reported data on TP, TN, FP, and FN cases per pCR definition were included for meta-analysis per imaging modality. With this information, two-by-two contingency tables were extracted per pCR definition. Positive was regarded as residual disease at final pathology or imaging and negative as either a pCR or an rCR. Subsequently, the pooled sensitivity and pooled specificity with corresponding 95% confidence intervals (95% CIs) were calculated separately for both definitions of pCR (i.e. ypT0 and ypT0/is). The heterogeneity among the included studies was explored using Cochran’s *Q* test and the inconsistency index (*I*^2^), with *p* < 0.05 or *I*^2^ > 50% indicating the presence of substantial heterogeneity. All statistical analyses were carried out using statistical software STATA (version 17.0; Stata Corp.).

## Results

### Study selection

**Fig. 1:**
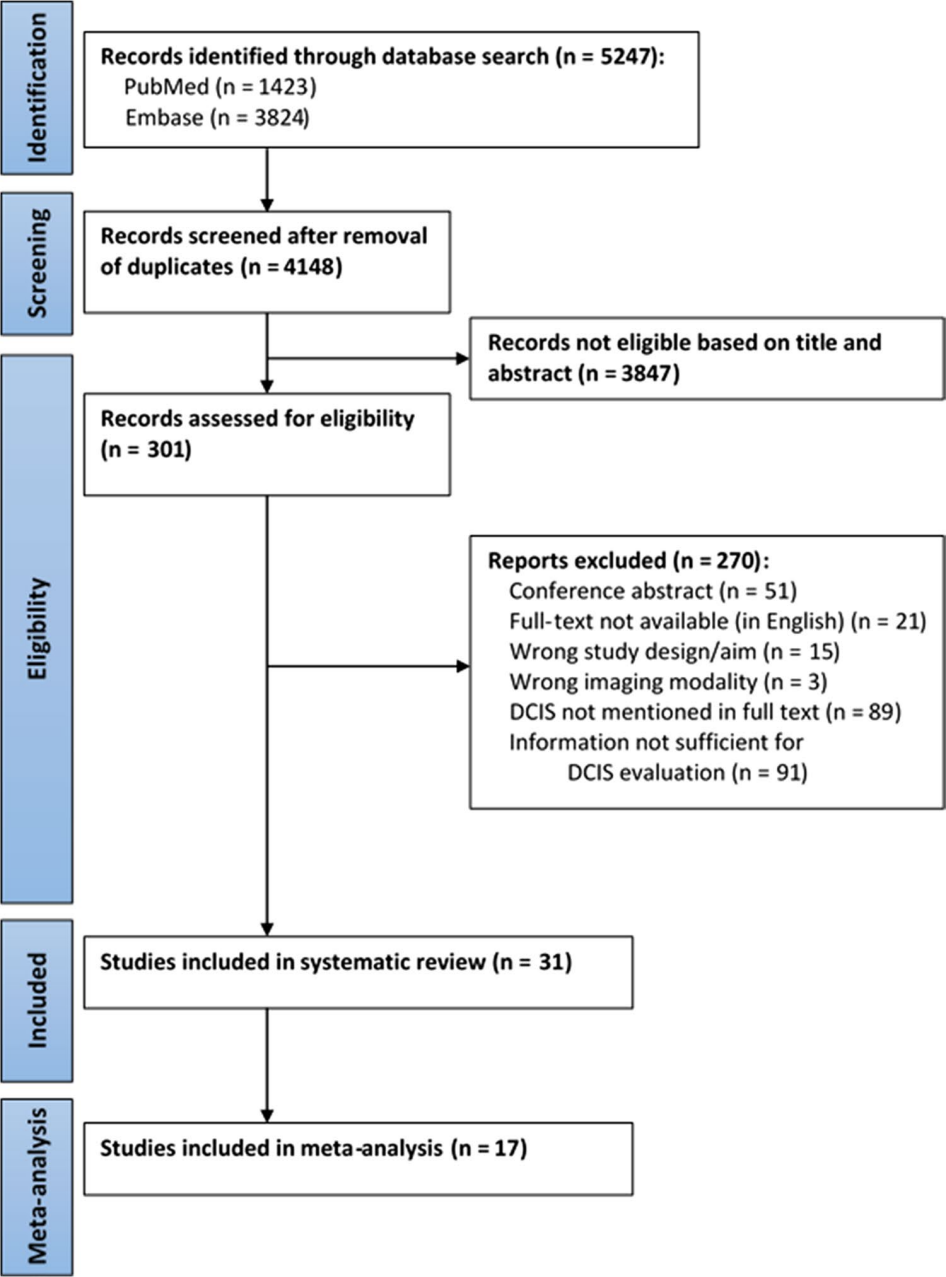
Flowchart of study selection

A total of 5247 studies were found by searching the Pubmed and Embase databases. After duplicates had been removed, titles and abstracts were screened, and 3847 studies were excluded as irrelevant. The remaining 301 full texts were read, and another 270 studies were excluded for various reasons (Fig. 1). Finally, 31 studies (4987 patients in total) were included in this systematic review, of which 17 were used for meta-analysis. Figure 1 includes a flowchart showing the literature selection. Table 1 provides an overview of included studies.

**Table 1:** Characteristics of included studies

Study (year)	Country	Study design	Patients	Imaging modality	pCR definition
Adrada (2015) [29]	USA	Retrospective	106	MG	ypT0
An (2017) [30]	Korea	Retrospective	29	MG	ypT0
Bernardi (2022) [31]	Italy	Prospective	51	MRI + CEM	ypT0
Bodini (2004) [32]	Italy	Prospective	73	MRI	ypT0^a^
Böttcher (2014) [33]	Germany	NR	54	MRI	ypT0
Chen (2008) [34]	USA	NR	51	MRI	ypT0/is
Choi (2012) [35]	Korea	Retrospective	46	MG + MRI	ypT0/is
De Los Santos (2011) [36]	USA	NR	81	MRI	ypT0 and ypT0/is
Feliciano (2017) [37]	USA	Retrospective	90	MG	ypT0
Gampenrieder (2019) [38]	Austria	Retrospective	246	MRI	ypT0/is N0
Goldberg (2017) [23]	Israel	Prospective	92	MG	ypT0
Groen (2021) [12]	Netherlands	Retrospective	316	MG + MRI	ypT0^a^
Hahn (2014) [39]	South Korea	Retrospective	78	MRI	ypT0/is
Hayashi (2013) [40]	Japan	NR	264	MRI	ypT0/is
Iotti (2017) [41]	Italy	Prospective	54	CEM	ypT0
Iotti (2021) [42]	Italy	Retrospective	36	CEM	ypT0 and ypT0/is
Iwase (2018) [43]	Japan	Retrospective	201	MRI	ypT0
Khazindar (2021) [44]	SAU	Retrospective	52	MRI	ypT0 and ypT0/is
Kim (2020) [45]	Korea	Retrospective	96	MG	ypT0
Lee (2017) [46]	USA	Prospective	30	MRI	ypT0
Li (2014) [47]	China	Retrospective	187	MG	ypT0 and ypT0/is
Mirza (2016) [48]	UK	NR	67	MRI	ypT0 and ypT0/is
Mistry (2015) [49]	India	Retrospective	446	MG	NR
Nakamura (2007) [50]	Japan	NR	115	MRI	ypT0
Negrão (2019) [51]	Brazil	Retrospective	219	MRI	ypT0/is
Park (2016) [52]	Korea	Retrospective	117	MG + MRI	ypT0/is
Santamaria (2019) [53]	Spain	Retrospective	81	MRI	ypT0 and ypT0/is
van Ramshorst (2017) [54]	Netherlands	Retrospective	330	MRI	ypT0/is
Vinnicombe (1996) [55]	UK	Retrospective	95	MG	ypT0^a^
Woodhams (2010) [56]	Japan	NR	69	MRI	ypT0^a^
Zhang (2020) [57]	China	Retrospective	1219	MRI	ypT0 and ypT0/is

*MG* mammography, *MRI* magnetic resonance imaging, *CEM* contrast-enhanced mammography, *NR* not reported

^a^Not reported, derived from text

### Quality of included studies

**Fig. 2:**
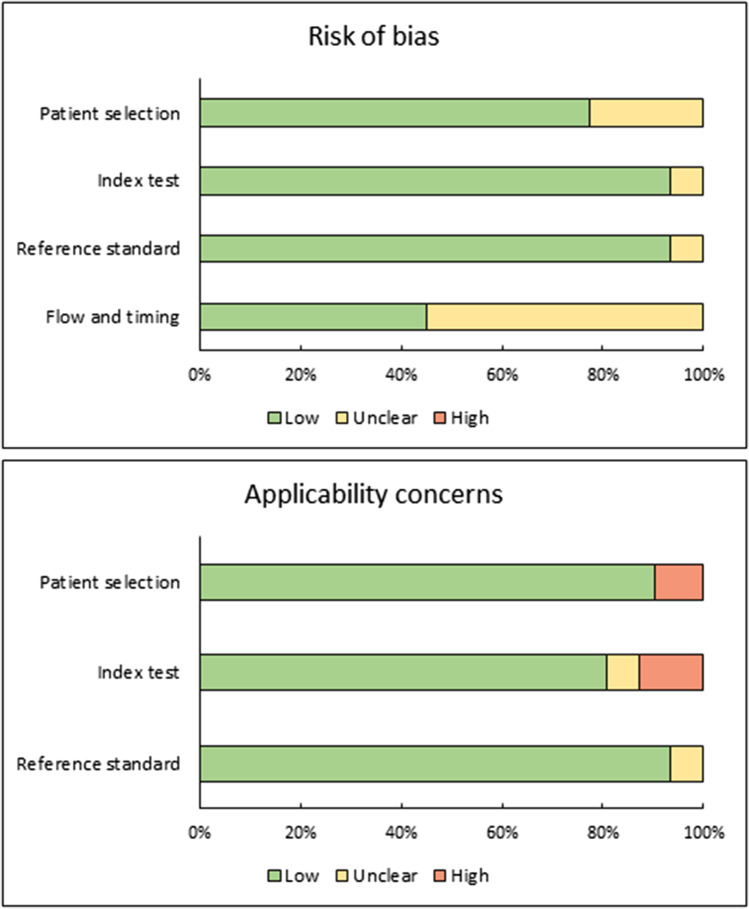
Summary of risk of bias and applicability concerns.

The results per category and study are reported in Table S3, and Fig. 2 summarises the risk of bias and applicability concerns. Overall, there was a low risk of bias regarding patient selection, index test, and reference standard. The risk of bias was often unclear for “flow and timing” because studies did not report time between imaging post-NST and surgery.

### Imaging findings of DCIS

Table 2 presents a summary of imaging findings pre- and post-NST per imaging modality. The characteristics of the imaging modalities, image evaluation, and the definitions used for rCR are reported in Table S4.

**Table 2:** Pre-NST and post-NST imaging findings per modality

Study (year)	Patients (***n***)	Pre-NST findings of DCIS	Post-NST findings of DCIS		
*Mammography*		*% of calcifications related to a DCIS component*	*% of calcifications related to a DCIS component*	*% of DCIS without calcifications*	*% of calcifications related to benign pathology*
Adrada (2015)	106	64.1%	29.2%	48.4%	40.6%
An (2017)	29	NR	34.5%	NR	44.8%
Choi (2012)	46	NR	45.8%	26.7%	54.2%
Feliciano (2017)	90	53.3%	36.7%	34%	62.2%
Groen (2021)	316	50.3%	NR	NR	NR
Kim (2020)	96	NR	50%	15.8%	38.5%
Li (2014)	187	NR	NR	NR	NR
Mistry (2016)	446	NR	60%	55.3%	NR
Vinnicombe (1996)	95	NR	42.1%	41.2%	NR
*Breast MRI*		*Pre-NST MRI findings of a DCIS component*	*Number of patients with ypTis*	*% ypTis with MRI enhancement*	*% ypTis without MRI enhancement*
Bernardi (2022)	51	NR	12	66.7%	33.3%
Bodini (2004)	73	NR	4	75%	25%
Böttcher (2014)	54	NR	6	33.3%	66.7%
Chen (2008)	51	NR	6	16.7%	83.3%
Choi (2012)	46	NR	15	93.3%	6.7%
De Los Santos (2011)	81	NR	9	66.7%	33.3%
Gampenrieder (2019)	246	NR	11	36.4%	63.6%
Hahn (2014)	78	NR	6	100%	0%
Hayashi (2013)	260	NR	32	78.1%	21.9%
Iwase (2018)	201	NR	14	64.3%	35.7%
Khazindar (2021)	52	NR	11	45.5%	54.5%
Lee (2017)	30	NR	2	0%	100%
Mirza (2016)	69	NR	6	33.3%	66.7%
Nakamura (2007)	115	NR	11	72.7%	27.3%
Negrão (2019)	219	NR	9	66.7%	33.3%
Park (2016)	117	NR	50	68%	32%
Santamaria (2019)	81	NR	8	75%	25%
Van Ramshorst (2017)	296	NR	69	23.2%	76.8%
Woodhams (2010)	69	NR	7	57.1%	42.9%
Zhang (2020)	1219	NR	60	68.3%	31.7%
*CEM*		*Pre-NST CEM findings of a DCIS component*	*Number of patients with ypTis*	*% enhancement in patients with ypTis*	*% calcifications in patients with ypTis*
Bernardi (2022)	51	NR	12	58.3%	NR
Iotti (2017)	46	NR	3	33.3%	NR
Iotti (2021)	36	NR	5	40%	100%

*DCIS* ductal carcinoma in situ, *NST* neoadjuvant systemic therapy, *MRI* magnetic resonance imaging, *CEM* contrast-enhanced mammography, *ypTis* residual DCIS in absence of residual invasive tumour, *NR* not reported

#### Pre-NST

Three mammography studies have reported imaging findings on pre-NST mammograms of patients with invasive breast cancer with a DCIS component [12, 29, 37]. More than half of the calcifications found on pre-NST mammography (50.3–64.1%) were related to a DCIS component.

Two studies on mammography and breast MRI investigated a study population of patients achieving pCR[ypT0/is] after NST [35, 52]. Pre-NST imaging findings of mammography and breast MRI were compared between the patients with ypT0 and ypTis: patients achieving ypTis more often had calcifications on mammography (54–87%) pre-NST compared to patients achieving ypT0 (16–35%). In addition, non-mass enhancement on breast MRI pre-NST was more frequent in patients achieving ypTis (28–80%) versus ypT0 (12–32%).

The remaining included breast MRI and CEM studies reported no imaging findings related to a DCIS component prior to NST.

#### Post-NST

Eight mammography studies have investigated the post-NST mammography findings of DCIS [29, 30, 35, 37, 45, 47, 49, 55]. Calcifications on mammography post-NST were related to DCIS (adjacent to IBC or residual DCIS only) in 29.2–60% [29, 30, 35, 37, 45, 49, 55]. Compared to patients with ypT0, patients with ypTis more often show calcifications on post-NST mammography (73.3% versus 41.9%) [35]. Of the DCIS components in the surgical specimen post-NST, 15.8–55.3% are not related to calcifications on mammography [29, 35, 37, 45, 49, 55]. In addition, 38.5–62.2% of calcifications post-NST were related to benign pathology [29, 30, 35, 37, 45]. Li et al [47] showed that calcifications post-NST outside the mass and calcifications that increased in size after NST had the highest percentage of ypTis (11.5% and 22.2%, respectively) [47].

The included breast MRI studies only described imaging findings of patients with ypTis rather than residual IBC with a DCIS component. Twenty breast MRI studies have investigated the percentage of patients with ypTis that showed enhancement on breast MRI (Table 2). The average percentage of ypTis that enhanced on MRI in these studies was 57.4% (200/348 patients) [31–36, 38–40, 43, 44, 46, 48, 50–54, 56, 57].

Two breast MRI studies demonstrated that ypTis was more frequently observed (68–93.3%) as residual disease on breast MRI post-NST compared to ypT0 (37–64.5%) [35, 52]. Choi et al found a significant correlation between residual DCIS size on breast MRI post-NST and histopathology (*r* = 0.81, *p* = 0.0003) [35].

The three CEM studies included showed the varying presentation of ypTis and discrepancy in comparison to MRI regarding enhancement [31, 41, 42]. The study by Iotti et al published in 2017 showed that MRI estimated the three patients with ypTis as complete response, while CEM showed residual enhancement in one [41]. The study by Bernardi et al showed that CEM demonstrated enhancement in 7 of 12 patients with ypTis, compared to 8 patients with enhancement on MRI [31]. The other study by Iotti et al published in 2021 demonstrated that on CEM, 3 out of 5 patients with ypTis had no residual enhancement, but all patients had residual pleiomorphic calcifications [42].

### Response evaluation of DCIS

Two studies have investigated the imaging findings of patients with response of DCIS [12, 23]. Goldberg et al investigated imaging findings of patients with response of DCIS on mammography. In their prospective cohort, 10 of 36 patients with a DCIS component pre-NST achieved pCR. In addition, 92% of calcifications remained on post-NST mammography despite complete response of the DCIS component [23].

Groen et al investigated mammography and breast MRI findings associated with response of DCIS. Mammography was performed only pre-NST and not post-NST. They defined rCR on MRI as no residual enhancement within the original tumour bed after NST and near complete response as only minimal residual enhancement in the original tumour bed, without any components clearly identifiable as original tumour. Multivariable logistic regression analyses reported absence of suspicious calcifications on pre-NST mammography (OR 3.51 (1.32–9.32)) and (near) complete response post-NST on breast MRI (OR 4.14 (1.36–12.59)) as independent factors associated with response of DCIS [12].

**Fig. 3:**
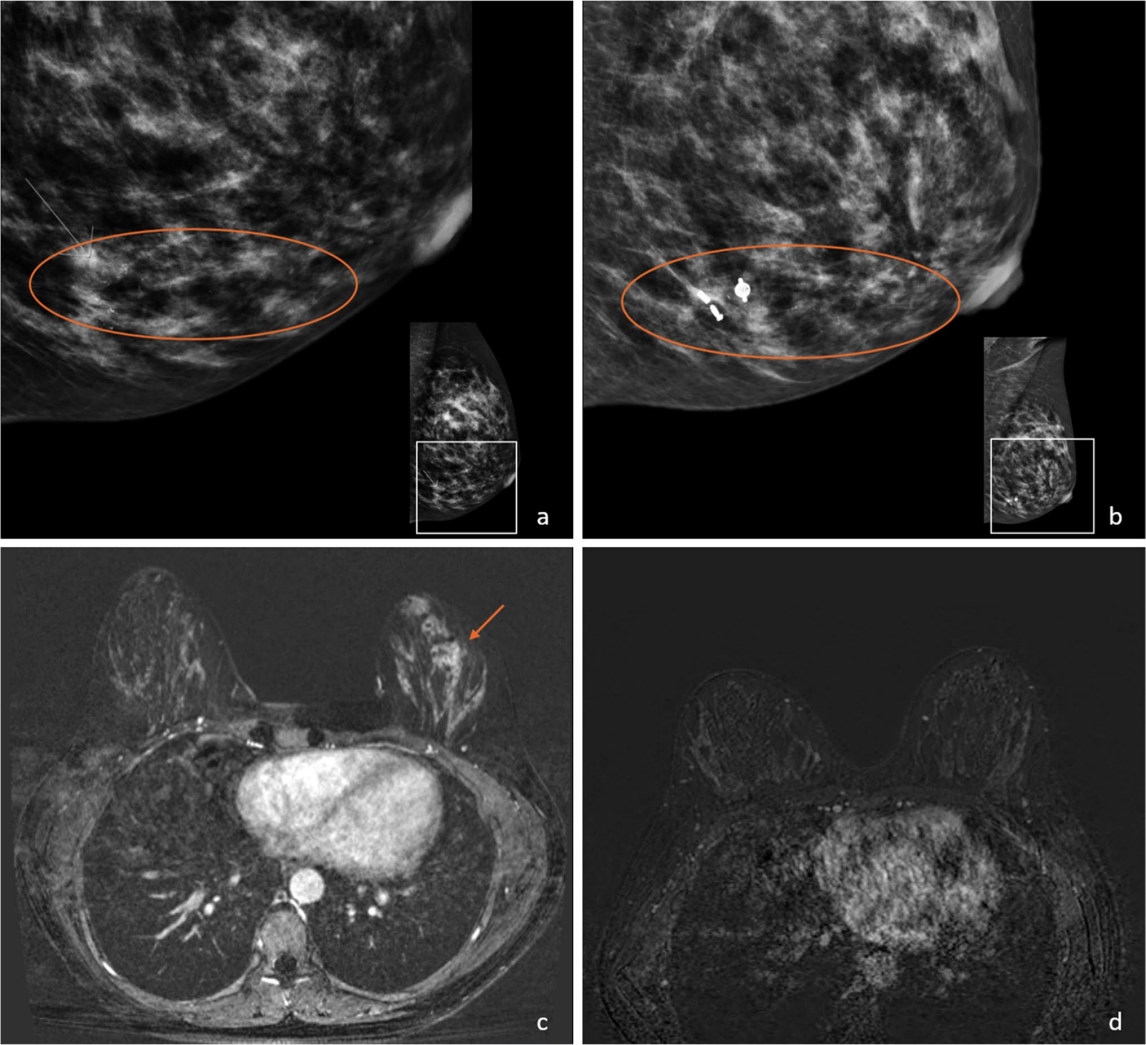
Pre- (**a**, **c**) and post-NST (**b, d**) mammography and MRI images of a patient with pCR of both IBC and DCIS. Fine pleiomorphic calcifications remained on mammography (**a**, **b**, orange circle), while NME (**c**, orange arrow) disappeared, classified as rCR on MRI

A clinical example of mammography and MRI images of a patient with pCR of both IBC and DCIS during NST is presented in Fig. 3. Mammography post-NST showed a persisting area of pleiomorphic calcifications, while on breast MRI post-NST, no persisting enhancement was found, classified as rCR.

### Meta-analysis of diagnostic performance in different pCR definitions

**Fig. 4:**
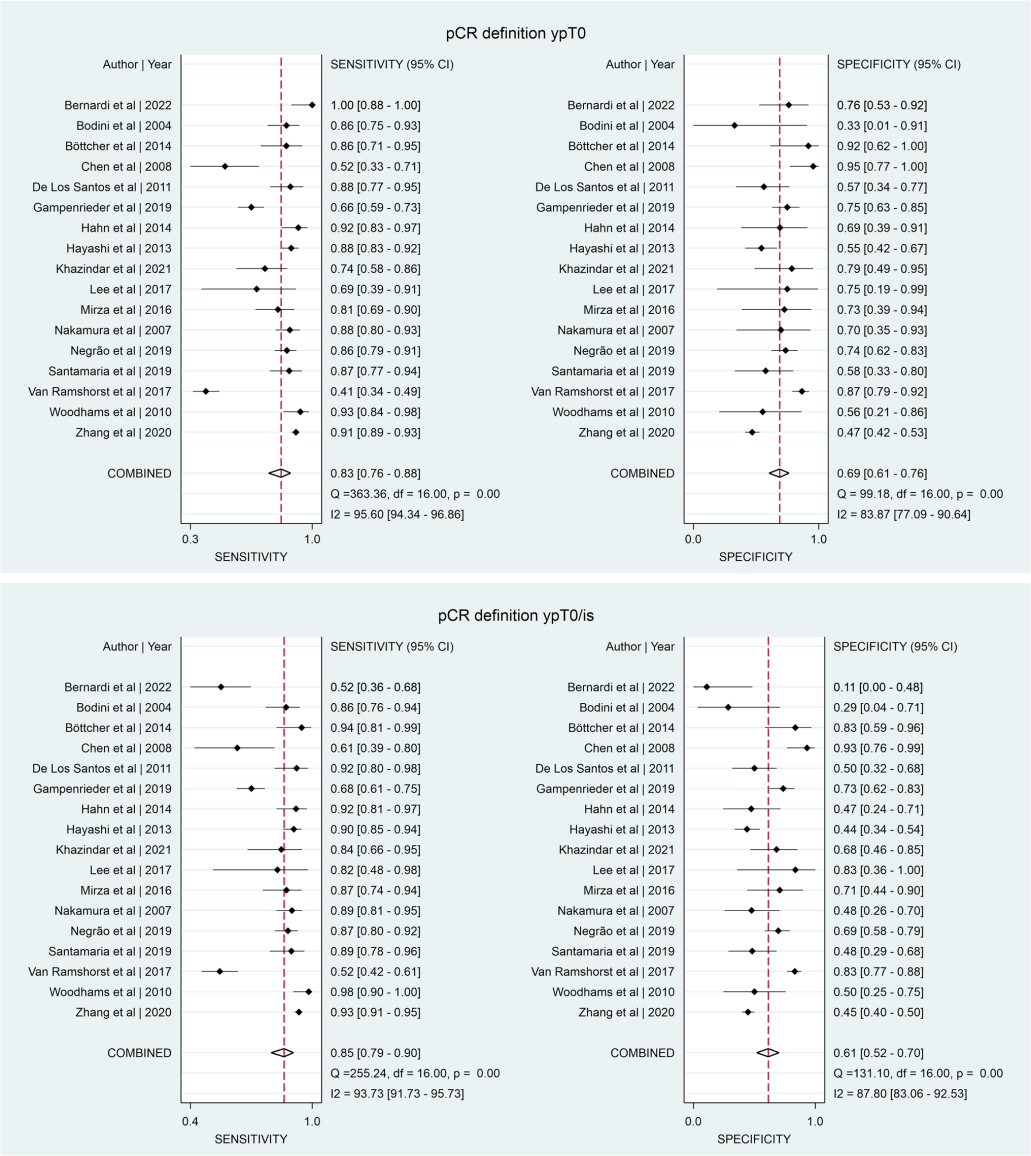
Pooled sensitivity and specificity for detection of residual disease by MRI between pCR defined as ypT0 and ypT0/is

Table 1 presents the definition of pCR used in the included studies. The mammography studies most frequently used ypT0 (7/11 studies) as pCR definition. In the MRI studies, 8 used ypT0/is, 8 used ypT0, and 5 used both definitions. In the 3 CEM studies included, two used ypT0 and one used both definitions. Seventeen breast MRI studies were included in the meta-analysis [31–34, 36, 38–40, 46, 48, 50, 51, 53, 54, 56, 57]. Meta-analyses for mammography and CEM could not be performed, due to a limited amount of studies including data on pCR definitions. In total, 787 patients in 17 studies achieved ypT0 and 269 patients had ypTis. Of the patients with ypTis, 143 out of 269 (53.2%) showed enhancement on MRI (Table S5). Figure 4 shows the pooled sensitivity and specificity per pCR definition. When ypT0/is is used, and DCIS is thus considered residual disease, sensitivity is slightly higher (0.85 versus 0.83) and specificity is lower (0.61 versus 0.69) compared to pCR defined as ypT0. There is a high heterogeneity in both groups, with *I*^2^ ranging from 84.2 to 95.6% (Fig. 4).

## Discussion

Since recent literature indicates potential response of DCIS to NST, accurate evaluation of both DCIS and IBC during NST is important for surgical planning. According to our knowledge, this is the first review to summarise the literature on response evaluation of the DCIS component in IBC patients with the imaging modalities mammography, breast MRI, and CEM. The 31 included studies did not specifically investigate imaging findings of DCIS response. Therefore, this review summarised additional information regarding pre- and post-NST imaging findings of a DCIS component and the influence of pCR definition on diagnostic performance. In general, we demonstrated that different findings per imaging modality are related to a DCIS component during NST.

On mammography, calcifications pre-NST are most often related to a DCIS component. In contrast, up to 50% of DCIS post-NST did not have calcifications and calcifications post-NST can remain without associated DCIS or IBC [29, 37]. Therefore, remaining calcifications should not generally be considered as residual DCIS but may represent a necrotic tumour bed in case of complete response of DCIS and IBC [58, 59]. Morphology of the calcifications can add important information to distinguish between malignant and benign findings. Previous mammographic studies have shown that fine-linear branching and fine pleiomorphic calcifications are most suspicious for high-grade DCIS with or without invasive breast cancer [15, 60, 61]. However, there were no studies correlating the morphology of calcifications to a DCIS component during NST.

On breast MRI, contrast enhancement of residual DCIS is varying. Overall in this review, 57% of the cases with residual DCIS (ypTis) demonstrated enhancement post-NST. There are a few possible explanations for the variable enhancement of residual DCIS. First, the sensitivity for detection of DCIS adjacent to IBC on MRI ranges between 39 and 84.9% [62, 63]. Another explanation might be the influence of the grade of DCIS on imaging findings. Previous literature showed that high-grade DCIS more often presents as an enhancing mass, while low-grade DCIS shows non-mass or no enhancement [17]. Moreover, MRI sensitivity is higher for high-grade DCIS than for low-grade DCIS (98% compared to 80%) [62]. It is important to note that, as presented in Table S4, the definition of rCR and the evaluation of enhancement differed between studies, which could have also contributed to the varying percentages of enhancement in patients with residual DCIS (Table 2).

No included studies described MRI findings of DCIS pre-NST. Considering a potential response of DCIS to NST, it is important to detect the DCIS component pre-NST and future studies should evaluate the change of imaging findings during NST compared to DCIS response.

Regarding CEM, only three studies were included in this review presenting information on a DCIS component. These three studies demonstrated a possible benefit of combining evaluation of calcifications and enhancement to detect residual DCIS. Compared to the other imaging modalities, CEM has been introduced more recently, and overall, less research has been conducted. Studies investigating CEM findings of pure DCIS described that enhancement and calcification features can contribute to differentiating between invasive breast cancer, DCIS, and benign lesions. Absence of enhancement in the presence of calcifications is mainly related to low-grade DCIS, although high-grade DCIS, like IBC, usually shows enhancement [64, 65]. This is in line with CEM studies in this systematic review in which part of the residual DCIS cases would have been missed on the basis of enhancement alone. The included CEM studies did not specify whether the evaluation of enhancement was based on objective or subjective assessment.

In general, most studies on NST response evaluation adhere to the pCR definition ypT0/is, thus considering residual DCIS as pCR. Our meta-analysis including 17 breast MRI studies demonstrated a slightly higher pooled sensitivity and a lower pooled specificity for detection of residual disease when DCIS was considered pCR (ypT0/is). This difference is explained by the higher numbers of false positives in pCR definition ypT0/is, because more than half of residual DCIS (53.2%) showed enhancement. A previous meta-analysis by Marinovich et al demonstrated similar results, with an increase in accuracy (i.e., a lower number of false positives and/or negatives) found when residual DCIS was excluded from the pCR definition [6]. However, it is important to emphasise that the differences in pooled sensitivity and specificity are small and that there is a high heterogeneity between included studies. More research is needed to investigate potential factors influencing the enhancement of residual DCIS, as this affects the diagnostic performance.

This meta-analysis further highlights the importance of distinguishing pCR from ypTis to establish true pCR. In our nationwide analysis of patients treated with NST, an average of 4.3% had ypTis, increasing up to 9.8% in HER2-positive invasive tumours [66]. Various clinical trials (e.g. NCT04578106) are investigating the possibility of omitting surgery in patients with expected pCR. Although no difference in prognosis between ypT0 and ypTis was reported in previous studies, it remains important to detect residual DCIS in these patients since this might cause positive surgical margins or even a recurrence of invasive cancer [67, 68]. Moreover, von Minckwitz et al demonstrated that ypT0/is has an increased risk of recurrence compared to ypT0 [69]. Current ongoing trials on fine-needle aspiration or vacuum-assisted core biopsies (NCT03188393, NCT02945579) investigate the potential of biopsies near the clip marker post-NST. However, an in situ component outside of the invasive tumour should be considered.

There are certain limitations to this review. First, there was a significant heterogeneity overall and per imaging modality. Between studies, the populations, imaging protocols, image evaluation, and study outcomes differed notably. Since this is the first review on this topic, this heterogeneity was expected in advance. Second, this review is influenced by the quality of the included studies, and despite an overall low risk of bias determined by the QUADAS-2 tool, there were applicability concerns for patient selection and the index test. Third, the results regarding the evaluation of the DCIS component during NST were often only described as secondary outcomes. Apart from the studies by Choi et al [35] and Park et al [52] aiming to distinguish between ypT0 and ypTis, the described imaging protocols of the included studies did not address detecting (residual) DCIS. For example, specific late phase enhancement evaluation, described as typical for DCIS in previous studies [26, 27], was only performed in two included studies [33, 53]. This makes some of the results difficult to interpret, and additional information on extent or imaging characteristics of DCIS were partially under-reported. However, due to the systematic approach of this review, we were able to summarise and evaluate the most important features of DCIS on imaging during NST. Future research should focus on DCIS adjacent to IBC to investigate possible influencing factors on diagnostic performance of imaging modalities.

In conclusion, different imaging findings on mammography, breast MRI, and CEM are related to a DCIS component. Most important to note is that residual calcifications do not necessarily indicate residual DCIS and that approximately 57% of residual DCIS shows enhancement on breast MRI. The meta-analysis shows a higher sensitivity and a lower specificity for detection of residual disease when DCIS is considered pCR (ypT0/is). Combining the imaging findings of calcifications and enhancement on CEM can be of potential benefit for evaluation of DCIS adjacent to IBC. This review provides a rationale for further research into imaging of DCIS adjacent to IBC during NST, given the current lack of evidence on imaging findings of response of the DCIS component.

## Supplementary Information

Below is the link to the electronic supplementary material.

Supplementary file1 (PDF 304 KB)

## Abbreviations

CEM: Contrast-enhanced mammography
DCIS: Ductal carcinoma in situ
FN: False negative
FP: False positive
IBC: Invasive breast cancer
NST: Neoadjuvant systemic therapy
pCR: Pathological complete response
TN: True negative
TP: True positive

## Declarations

### Guarantor

The scientific guarantor of this publication is T.J.A. van Nijnatten.

### Statistics and biometry

One of the authors has significant statistical expertise.

### Methodology

- Performed at one institution
