# Supplementary material for: Imaging findings for response evaluation of ductal carcinoma in situ in breast cancer patients treated with neoadjuvant systemic therapy: a systematic review and meta-analysis
Source: Eur Radiol. 2023 Apr 5;33(8):5423–35. doi: 10.1007/s00330-023-09547-7 (PMC10326113; doi:10.1007/s00330-023-09547-7)
Supplement: Supplementary file 1 — Supplementary file1 (PDF 304 KB) [file 330_2023_9547_MOESM1_ESM.pdf]

Supplemental 1: Pubmed search

("breast neoplasms"[MeSH Terms] OR (("breast"[MeSH Terms] OR "breast*"[Title/Abstract] OR "mamma*"[Title/Abstract]) AND ("neoplas*"[Title/Abstract] OR "tumor*"[Title/Abstract] OR "tumour*"[Title/Abstract] OR "cancer*"[Title/Abstract] OR "malign*"[Title/Abstract] OR "carcinom*"[Title/Abstract])) OR ("carcinoma, intraductal, noninfiltrating"[MeSH Terms] OR "ductal carcinoma in situ"[Title/Abstract] OR "DCIS"[Title/Abstract] OR "intraductal carcinom*"[Title/Abstract])) AND ("magnetic resonance imaging"[MeSH Terms] OR "magnetic resonance imaging"[Title/Abstract] OR "MRI"[Title/Abstract] OR "magnetic resonance image"[Title/Abstract] OR "nmr imaging"[Title/Abstract] OR "mr tomography"[Title/Abstract] OR "nmr tomography"[Title/Abstract] OR ("mammography"[MeSH Terms] OR "digital breast tomosynthesis"[Title/Abstract] OR "digital mammograph*"[Title/Abstract] OR "mammograph*"[Title/Abstract]) OR ("contrast media"[MeSH Terms] OR "contrast enhanced mammography"[Title/Abstract] OR "CESM"[Title/Abstract] OR "contrast enhanced spectral mammography"[Title/Abstract])) AND ("neoadjuvant therapy"[MeSH Terms] OR "neoadjuvant chemotherapy"[Title/Abstract] OR "neoadjuvant chemotherapy treatment"[Title/Abstract] OR "neoadjuvant systemic therapy"[Title/Abstract] OR "neoadjuvant systemic treatment"[Title/Abstract] OR "neoadjuvant treatment"[Title/Abstract] OR "neoadjuvant endocrine therapy"[Title/Abstract] OR "neoadjuvant targeted therapy"[Title/Abstract] OR (("hormone therapy"[Title/Abstract] OR "endocrine therapy"[Title/Abstract] OR "targeted therapy"[Title/Abstract]) AND "Neoadjuvant"[Title/Abstract]) OR "preoperative chemotherapy"[Title/Abstract])

Supplemental 2: Embase search

1	breast tumor/	24	CESM.ti,ab,kw.
2	breast/	25	22 or 23 or 24
3	breast*.ti,ab,kw.	26	nuclear magnetic resonance imaging/
4	mamma*.ti,ab,kw.	27	magnetic resonance imaging*.ti,ab,kw.
5	2 or 3 or 4	28	MRI*.ti,ab,kw.
6	malignant neoplasm/	29	26 or 27 or 28
7	(neoplas* or tumor* or tumour* or cancer* or malign* or carcinom*).ti,ab,kw.	30	21 or 25 or 29
8	6 or 7	31	exp neoadjuvant therapy/
9	5 and 8	32	preoperative treatment/
10	1 or 9	33	preoperative chemotherapy/
11	exp intraductal carcinoma/	34	31 or 32 or 33
12	intraductal carcinom*.ti,ab,kw.	35	(neoadjuvant* or neo adjuvant* or preoperative* or pre operative*).ti,ab,kw.
13	ductal carcinoma in situ.ti,ab,kw.	36	chemotherapy/
14	DCIS.ti,ab,kw.	37	systemic therapy/
15	11 or 12 or 13 or 14	38	immunotherapy/
16	10 or 15	39	molecularly targeted therapy/
17	exp mammography/	40	antineoplastic agent/
18	exp digital mammography/	41	(chemotherap* or systemic therap* or immunotherap* or targeted therap* or antineoplastic).ti,ab,kw.
19	mammograph*.ti,ab,kw.	42	36 or 37 or 38 or 39 or 40 or 41
20	digital breast tomosynthes*.ti,ab,kw.	43	35 and 42
21	17 or 18 or 19 or 20	44	34 or 43
22	contrast enhanced spectral mammograph*.ti,ab,kw.	45	16 and 30 and 44
23	contrast enhanced mammograph*.ti,ab,kw.		

Supplemental 3

Table S3 Quadas-2 classification

Study	Risk of bias				Applicability concerns		
	Patient selection	Index test	Reference standard	Flow and timing	Patient selection	Index test	Reference standard
Adrada (2015)[27]	Low	Low	Low	Unclear	Low	Low	Low
An (2017)[32]	Low	Low	Low	Unclear	Low	Low	Low
Bernardi (2022)[54]	Low	Low	Low	Unclear	Low	Low	Low
Bodini (2004)[37]	Low	Low	Low	Unclear	Low	High	Low
Böttcher (2014)[38]	Unclear	Low	Low	Low	Low	Low	Low
Chen (2008)[39]	Unclear	Low	Low	Low	Low	Low	Low
Choi (2012)[30]	Low	Low	Low	Low	High	Low	Low
De Los Santos (2011)[40]	Unclear	Low	Low	Unclear	Low	Low	Low
Feliciano (2017)[28]	Low	Low	Low	Unclear	Low	Low	Low
Gampenrieder (2019)[41]	Low	Low	Low	Low	Low	High	Low
Goldberg (2017)[23]	Low	Unclear	Low	Unclear	Low	Unclear	Low
Groen (2021)[29]	Low	Low	Low	Unclear	Low	Low	Low
Hahn (2014)[42]	Low	Low	Low	Low	Low	Low	Low
Hayashi (2013)[43]	Unclear	Low	Low	Unclear	Low	Low	Low
Iotti (2017)[55]	Low	Low	Low	Unclear	Low	Low	Low
Iotti (2021)[56]	Low	Low	Low	Unclear	Low	Low	Low
Iwase (2018)[44]	Low	Low	Low	Unclear	Low	High	Low
Khazindar (2021)[45]	Low	Low	Low	Unclear	Low	Low	Low
Kim (2020)[33]	Low	Low	Low	Unclear	Low	Low	Low
Lee (2017)[46]	Low	Low	Low	Low	Low	Low	Low
Li (2014)[34]	Low	Low	Low	Low	Low	Low	Low
Mirza (2016)[47]	Unclear	Low	Low	Low	Low	Low	Low
Mistry (2015)[35]	Low	Low	Low	Low	Low	Low	Low
Nakamura (2007)[48]	Unclear	Unclear	Unclear	Unclear	High	Unclear	Unclear
Negrão (2019)[49]	Low	Low	Low	Unclear	Low	Low	Low
Park (2016)[31]	Low	Low	Low	Unclear	High	Low	Low
Santamaria (2019)[50]	Low	Low	Low	Low	Low	Low	Low
van Ramshorst (2017)[51]	Low	Low	Low	Low	Low	Low	Low
Vinnicombe (1996)[36]	Low	Low	Unclear	Low	Low	High	Unclear
Woodhams (2010)[52]	Unclear	Low	Low	Low	Low	Low	Low
Zhang (2020)[53]	Low	Low	Low	Low	Low	Low	Low

Supplemental 4: Table S4: Imaging modality, protocol, and image evaluation

Study	Patients (n)	Modality unit	Modality vendor	Time period of scans	Imaging protocol	Image evaluation	Definition of rCR
Mammography							
Adrada (2015)[27]	106	1. LORAD M3<br>2. DMR	1. Hologic<br>2. GE Healthcare	January 2004 - December 2008	Standard three-view diagnostic mammography	Pre-NST compared to post-NST	NR
An (2017)[32]	29	1. LORAD Selenia<br>2. Mammomat Inspiration	1. Hologic<br>2. Siemens Healthineers	April 2015 - April 2016	Craniocaudal and mediolateral oblique mammograms	Pre-NST compared to post-NST	NR
Choi (2012)[30]	46	1. Senographe 2000D<br>2. LORAD Selenia	1. GE Healthcare<br>2. Hologic	January 2006 - December 2008	Craniocaudal and mediolateral oblique mammograms	Pre-NST compared to post-NST	NR
Feliciano (2017)[28]	90	NR	NR	April 2009 - October 2015	Craniocaudal and mediolateral oblique mammograms	Pre-NST compared to post-NST	NR
Goldberg (2017)[23]	92	NR	NR	March 2011 - November 2013	NR	Pre-NST compared to post-NST	NR
Groen (2021)[29]	316	NR	NR	January 2004 - November 2017	NR	Only pre-NST mammography	NR
Kim (2020)[33]	96	LORAD Selenia	Hologic	June 2015 - August 2018	Craniocaudal and mediolateral oblique mammograms	Pre-NST compared to post-NST	NR
Li (2014)[34]	187	NR	NR	February 2008 - March 2013	Standard four view film/screen mammography	Pre-NST compared to post-NST	NR
Mistry (2016)[35]	446	Senographe DS	GE Healthcare	January 2007 - December 2011	NR	Pre-NST compared to post-NST	No measurable mass
Park (2016)[31]	117	NR	NR	January 2010 - December 2013	NR	Pre-NST compared to post-NST	No residual mass or calcifications
Vinnicombe (1996)[36]	95	NR	NR	NR	Craniocaudal and mediolateral oblique mammograms	Pre-NST compared to post-NST	Complete resolution of any mass with no residual abnormality at mammography
Breast MRI							
Bernardi (2022)[54]	51	Signa (1.5T)	GE Healthcare	May 2015 - April 2018	Three examinations per patient: an axial T2-weighted STIR sequence (TR/TE, 5362/50 ms; inversion time, 150 ms; FA, 160°; slice thickness, 2 mm; matrix, 356 × 356), an axial DWI echo-planar sequence (TR/TE, 8883/70 ms; slice thickness, 3	Use of subtraction images: yes<br>ROI: NR<br>CAD: NR<br>Enhancement evaluation: subjective	If the lesion was no longer visualized

					mm; matrix, 356 × 356; b values, 50 and 800 s/mm2), and an axial T1-weighted gradient-echo 3D VIBRANT-Flex sequence (TR/TE, 6.6/4.5 ms; slice thickness, 1.6 mm; matrix, 356 × 356) acquired once before and five times after IV contrast administration.	Specific evaluation of late enhancement: no	
Bodini (2004)[37]	73	Gyroscan NST (0.5T)	Philips Healthcare	January 1998 - August 2001	The sequences used were T1 weighted 3D-FFEs, optimized to last less than 90 s and to cover the entire mammary gland and partially the axillary cavity with contiguous scans of 2.7–3 mm. More precisely, the following parameters were used: TR = minimum possible (23 ms); TE = 13.8 ms (in phase for 0.5 T unit); FA = 40◦; partition = 40 (or up to 48); slice thickness 2.7–3 mm; FOV = 340; RFOV = 45–50%; matrix = 60%. The same sequence was repeated before and, with no interval between repeats, five times 10 s after contrast injection.	Use of subtraction images: yes<br>ROI: manually<br>CAD: yes<br>Enhancement evaluation: subjective and objective (intensity/time curves)<br>Specific evaluation of late enhancement: no	Complete resolution of both tumour mass and enhancement
Böttcher (2014)[38]	54	Achieva (1.5T)	Philips Healthcare	NR	Three-dimensional gradient-echo T1-weighted axial sequences were acquired for dynamic imaging with the following parameters: TR 8.3 ms, TE 4.6 ms, FA 12 °, FOV 320 – 360 mm, matrix 360 x 360, spatial resolution 0.7 x 0.7 x 2 mm 3. Axial T2-weighted turbo spin-echo sequences were performed before contrast medium application (TR 4618 ms, TE 120 ms, FA 90 °, FOV 320 – 360 mm, matrix 448 x 330, spatial resolution 0.7 x 0.7 x 3 mm 3). Twenty seconds after the beginning of contrast medium injection, dynamic scanning was performed with the same sequence parameters and under identical tuning conditions; five post-contrast series were acquired with identical time intervals of 58s.	Use of subtraction images: yes<br>ROI: manually<br>CAD: yes<br>Enhancement evaluation: subjective and objective (intensity time curves, maximum signal intensity, morpho-dynamic index)<br>Specific evaluation of late enhancement: yes	According to complete response in the RECIST guidelines
Chen (2008)[39]	51	NR (1.5T)	Philips Healthcare	July 2003 - April 2006	Imaging protocol consisted of high-resolution precontrast imaging and	Use of subtraction images: yes	Cases in which no enhanced tissues

					dynamic contrast-enhanced imaging. After a scout scan, sagittal, unilateral T1-weighted, precontrast images were acquired. After this, a 3D Spoiled Gradient Recalled (SPGR) pulse sequence with 16 frames, including 4 precontrast and 12 postcontrast sets, was prescribed for axial, bilateral, dynamic imaging (TR = 8.1 ms, TE = 4.0ms, FA = 20°, slice thickness = 4 mm, matrix size = 256 by 128, FOV = 32–38 cm). The scan time was 42 seconds per acquisition. A contrast agent was injected the beginning of the fifth acquisition	ROI: automatic<br>CAD: yes<br>Enhancement evaluation: subjective and objective (colour coded mapping, maximum intensity projections)<br>Specific evaluation of late enhancement: no	were visible and cases with minimal enhancement found at the previous lesion with weaker or comparable enhancement relative to normal glandular tissue.
Choi (2012)[30]	46	Signa (1.5T)	GE Healthcare	January 2006 - December 2008	Fat suppressed T2-weighted fast spin echo sagittal images were obtained using TR 5500 ms/TE 85.2 ms, FA, 90◦; image matrix, 256 × 160; FOV, 200 mm × 200 mm; and slice thickness/gap, 1.5 mm/0 mm. A three dimensional, T1-weighted fast spoiled gradient-echo (SPGR) sequence was also performed with bilateral sagittal scanning, with one pre-contrast and four post-contrast dynamic series after 90 s, 270 s, 360 s and 510 s. Image parameters were: TR ms/TE ms, 6.5/2.5; FA, 20◦; image matrix, 320 × 160; FOV, 200 mm × 200 mm; and slice thickness/gap, 1.5 mm/0 mm.	Use of subtraction images: yes<br>ROI: manually<br>CAD: NR<br>Enhancement evaluation: subjective and objective (time-intensity curves and fibroglandular signal intensity ratio)<br>Specific evaluation of late enhancement: no	No enhancing lesion
De Los Santos (2011)[40]	81	NR (1.5T)	GE Healthcare	January 2002 - February 2009	Sequences were as follows: T1 axial, T2 fat-sat axial, pre- and post-contrast sagittal dynamic sequence with four repeats at 90-second intervals, 2- to 3-mm slice thickness, and delayed post-contrast T1 fat-sat axial, colour map analysis, and maximum intensity projection	Use of subtraction images: NR<br>ROI: NR<br>CAD: yes<br>Enhancement evaluation: subjective<br>Specific evaluation of late enhancement: no	Resolution of the mass and absence of suspicious enhancement on post-treatment scan
Gampenrieder (2019)[41]	246	1. Philips Achieva<br>2. Philips Ingenia	Philips Healthcare	September 2006 - May 2016	We obtained axial T2-weighted fat-suppressed images (TE/TR/IR of 60/9065/230 msec; with a slice thickness of 3 mm and a FOV of 30–40 cm) and axial diffusion-weighted images (TE IR/TR	Use of subtraction images: NR<br>ROI: NR<br>CAD: NR	Absence on visual inspection of contrast enhancement on any serial image of dynamic contrast-

		(3.0T, 1.5T and 11 patients on <1.5T)			of 59/8157 msec; b-values up to 600; slice thickness 3 mm). Finally, four axial T1-weighted fat-suppressed dynamic acquisitions (TE/TR, 2.3/4.1 msec with a slice thickness of 1 mm) were registered over the duration of 5 min after intravenous contrast medium injection	Enhancement evaluation: subjective<br>Specific evaluation of late enhancement: no	enhanced T1-weighted MRI
Groen (2021)[29]	316	NR	NR	January 2004 - November 2017	NR	NR	No residual enhancement within the original tumour bed after NST
Hahn (2014)[42]	78	1. Signa (1.5T)<br>2. Achieva (3.0T)	1. GE Healthcare<br>2. Philips Healthcare	July 2008 - December 2009	MRI consisted of a fat-suppressed axial fast spin echo T2-weighted sequence and a dynamic axial three-dimensional T1-weighted fast spoiled gradient-echo sequence. Pre-contrast images for dynamic enhancement were obtained just before injection of the contrast agent, and the sequential post-contrast images were obtained every minute for 6 min with no delay at the same slice position and location. Imaging on the 1.5 T scanner covered both breasts with minimum TR and TE (6.5/2.5), a 10◦ FA, a 300 mm FOV, 1.5 mm sections with no gap, a 376 × 374 matrix, and a scan time of approximately 60 s. Imaging on the 3.0 T scanner covered both breasts with minimum TR and TE (5.0/2.5), a 12◦ FA, a 300 mm FOV, 1.5 mm sections with no gap, a 376 × 374 matrix, and a scan time of approximately 60 s. Following contrast material injection, axial delayed-phase imaging in bilateral breasts was performed at 450 s after injection	Use of subtraction images: NR<br>ROI: manually<br>CAD: NR<br>Enhancement evaluation: subjective<br>Specific evaluation of late enhancement: no	NR
Hayashi (2013)[43]	260	Intera Achieva Nova Dual (1.5T)	Philips	February 2003 - June 2008	Transverse images were obtained by DWI. Coronal images were obtained by contrast-enhanced dynamic imaging. Sagittal images were obtained by contrast-enhanced late-phase imaging. Additionally, depending on the case, sagittal images were obtained by T2-	Use of subtraction images: NR<br>ROI: NR<br>CAD: NR<br>Enhancement evaluation: subjective	No gadolinium enhancement or an enhancement equal to or less than that of glandular tissue in any phase of the MRI.

					weighted fat-suppressed imaging prior to infusion of contrast material. Dynamic phase consisted of 1 pre-contrast scan and 5 post-contrast scans. Details for dynamic study: TR 11 ms, TE 5.6ms, FA 20◦, scan time 30.2 sec/phase	Specific evaluation of late enhancement: no	
Iwase (2018)[44]	201	Signa (3.0T)	GE Healthcare	January 2013 - November 2016	The imaging protocol consisted of pre-contrast and dynamic contrast enhanced imaging. Dynamic study was performed before and 45, 180, and 315 s after starting intravenous gadolinium injection.	Use of subtraction images: NR<br>ROI: NR<br>CAD: NR<br>Enhancement evaluation: subjective<br>Specific evaluation of late enhancement: no	No enhanced lesion on MRI, and US findings would also support it, or if the enhanced lesions on MRI were not confirmed on US.
Khazindar (2021)[45]	52	Skyra (3.0T)	Siemens Healthineers	January 2016 - January 2019	NR	NR	Resolution of all areas of abnormal enhancement, mass, or distortion
Lee (2017)[46]	30	1. Excite HD (1.5T)<br>2. Symphony (1.5T)	1. GE Healthcare<br>2. Siemens Healthineers	January 2014 - October 2015	The MRI protocol included axial and coronal short-tau inversion recovery images, a pre-contrast T1-weighted acquisition, and additional postcontrast T1-weighted acquisitions obtained up to 5 to 7 minutes after the administration of the contrast agent.	Use of subtraction images: yes<br>ROI: manually<br>CAD: yes<br>Enhancement evaluation: subjective and objective (time intensity curves, highest tumour enhancement)<br>Specific evaluation of late enhancement: no	NR
Mirza (2016)[47]	67	Symphony (1.5T)	Siemens Healthineers	NR	Dynamic acquisition of images with one pre-contrast and five post-contrast measurements in the axial plane after intravenous bolus injection of contrast agent. Technical details: T1 gradient echo sequence with fat suppression, TR 4.42, TE 1.63, slice thickness 1.2mm/20%gap, FOV 320, base resolution 512, phase resolution 66%, slice resolution 60%, partial fourier 6/8, voxel size 0.9 x 0.6 x 1.2.	Use of subtraction images: yes<br>ROI: NR<br>CAD: NR<br>Enhancement evaluation: subjective<br>Specific evaluation of late enhancement: no	Complete disappearance of tumor mass and malignant enhancement in the breast and axilla on the post-NAC MR image
Nakamura (2007)[48]	115	NR	NR	NR	NR	NR	No visible enhancement on

							MRM or only fibrous changes without enhancement
Negrão (2019)[49]	219	Achieva (1.5T)	Philips Healthcare	October 2014 - July 2017	Prior to administration of contrast, T1 gradient-echo phase, 3D imaging was acquired in the axial plane (TR/TE, 627/8.0 ms; 3 mm-thick slices; 280 × 399 matrix; FOV, 250 mm). A fat-saturated short tau inversion recovery (STIR) sequence in the sagittal plane of both breasts was also acquired (TR/TE, 5127/80 ms; 3 mm-thick slices; 220 × 208 matrix; FOV, 220 mm). Five gradient-echo phases in T1, 3D, and in the axial plane were further obtained by using fat suppression for dynamic examination (TR/TE, 5.1/2.5 ms; 1 mm-thick slices; 352 × 429 matrix; FOV, 300 mm). The first phase was obtained prior to the injection of contrast reagent, the second phase was obtained 20 s after the injection of contrast agent, and another phase was obtained in the subsequent minutes with a temporal resolution of 60–90 s. The last sequence consisted of a sagittal T1-weighted, 3D gradient-echo pulse sequence with fat signal suppression (TR/TE, 5.5/2.9 ms; 1 mm-thick slices; 368 × 364 matrix; FOV, 220 mm).	Use of subtraction images: yes<br>ROI: NR<br>CAD: NR<br>Enhancement evaluation: subjective<br>Specific evaluation of late enhancement: no	Absence of enhancement in the topography of the previous lesion or metallic clip, or when enhancement was equal to or lesser than the normal breast tissue
Park (2016)[31]	117	NR	NR	January 2010 - December 2013	NR	Use of subtraction images: yes<br>ROI: NR<br>CAD: yes<br>Enhancement evaluation: subjective and objective (time-intensity curve)<br>Specific evaluation of late enhancement: no	Absence of a distinct enhancing area
Santamaria (2019)[50]	81	1. Signa (1.5T)<br>2. Aera (1.5T)	1. GE Healthcare<br>2. Siemens Healthineers	January 2015 - June 2017	Pretreatment and posttreatment MR imaging were performed in the same magnet for each patient. 3D T1 weighted	Use of subtraction images: NR<br>ROI: NR	Complete absence of both early and late enhancement

					sequence of SIGNA MRI: TR 4.7, TE 2.3, FA 15◦, slice thickness 2, FOV 330, matrix 416 x 416, acquisition time 72s. 3D T1 weighted sequence of AERA MRI: TR 4.65, TE 1.78, FA 10◦, slice thickness 2, FOV 340, matrix 416 x 416, acquisition time 75x.	CAD: NR<br>Enhancement evaluation: subjective<br>Specific evaluation of late enhancement: yes	
Van Ramshorst (2017)[51]	330	1. Magnetom Vision (1.5T)<br>2. Achieva (3.0T)	1. Siemens Healthineers<br>2. Philips Healthcare	January 2000 - March 2016	First, an unenhanced coronal 3D fast field echo (thrive) sense T1-weighted sequence was performed. Subsequently, contrast was administered intravenously followed by dynamic imaging in five consecutive series at 90-s intervals with voxel size 1.21 9 1.21 9 1.69 mm3 (1.5 T) or 1.1 9 1.1 9 1.2 mm3 (3.0 T)	Use of subtraction images: NR<br>ROI: NR<br>CAD: NR<br>Enhancement evaluation: subjective<br>Specific evaluation of late enhancement: no	Absence of pathologic (i.e. non-physiological) contrast enhancement in the original tumour region
Woodhams (2010)[52]	69	HDx (1.5T)	GE Healthcare	January 2005 - November 2008	After DWI, unilateral examination of the index breast was performed and consisted of the following: (a) a sagittal T2-weighted fast spin-echo sequence with fat suppression, TR/TE 4000ms/90ms, FOV 200mm, matrix 288 x 192, section thickness 5mm, and (b) a dynamic sagittal three-dimensional T1-weighted fast spoiled gradient-echo sequence, TR/TE 16.3ms/2.1ms, FOV 200, matrix 288 x 192, section thickness 2.0mm. For dynamic contrast enhancement, acquisitions were obtained before contrast agent injection and at 90 seconds (early phase) with centric encoding and at 300 seconds (delayed phase) after bolus injection. Following contrast material injection, axial delayed-phase imaging in bilateral breasts was performed at 450 seconds after injection.	Use of subtraction images: NR<br>ROI: manually<br>CAD: NR<br>Enhancement evaluation: subjective<br>Specific evaluation of late enhancement: no	NR
Zhang (2020)[53]	1219	Aurora Imaging Technology (1.5T)	Aurora Systems	November 2013 - March 2018	The following sequences were acquired while patients were in a prone position: a pre-contrast axial T2-weighted fat-suppressed sequence (TR 6,680 ms, TE 29 ms, thickness 3 mm) and axial T1-weighted fat-suppressed sequences (TR	Use of subtraction images: NR<br>ROI: NR<br>CAD: NR<br>Enhancement evaluation: subjective	No enhanced tumor visible on any serial images of dynamic contrast-enhanced T1-weighted images

					4.8 ms, TE 29 ms, thickness 1.1 mm, FOV 360 mm, matrix 360×360×128) before and after a bolus of a gadolinium-based contrast agent was injected at a rate of 2 mL/s. Postcontrast images were obtained at 90, 180, 270, and 360 seconds after the injection	Specific evaluation of late enhancement: no	
CEM							
Bernardi (2022)[54]	51	Selenia Dimensions	Hologic	May 2015 - April 2018	For pre-NST CEM examinations, two standard craniocaudal and mediolateral oblique views were acquired of each breast. For mid-NST and post-NST examinations, CEM was performed only of the affected breast to reduce radiation exposure for study participants. In addition, for post-NST examinations, a delayed CEM acquisition of the affected breast was obtained at 6 minutes after contrast material injection. A 6-minute interval was used for the delayed CEM acquisition.	Image analyses performed on recombined images based on contrast uptake (subjective).<br><br>Specific evaluation of late enhancement: yes	If the lesion was no langer visualized
Iotti (2017)[55]	54	Senographe Essential	GE Healthcare	October 2012 - December 2014	After contrast administration, a set of low-energy and high-energy images is acquired in quick succession while the breast remains compressed, obtaining a low-dose image, comparable to a standard digital mammogram, and a post-processing recombined image, which enhances the distribution of the iodine contrast medium. In a monolateral CEM, the radiographer compressed the breast for the mediolateral oblique projection 2 minutes after administration of contrast agent and then decompressed the breast, and after a further 2 minutes again compressed the breast for the cranio-caudal projection.	Image analyses performed on recombined images, based on contrast uptake (subjective).<br><br>Specific evaluation of late enhancement: no	Disappearance of all lesions
Iotti (2021)[56]	36	NR	GE Healthcare	2012-2020	After contrast administration, a low- and a high-energy image for each breast standard projection are acquired in quick succession while the breast remains	Image analyses performed on recombined images, based on contrast uptake (subjective).	Absence of residual disease based on absence of contrast enhancement and

					compressed; the low-dose image obtained is comparable to a standard digital mammogram, and the post-processing recombined image enhances the distribution of the iodine contrast medium.	Specific evaluation of late enhancement: no	absence of malignant calcifications (fine pleomorphic, coarse heterogeneous, fine linear/branching, etc)

Abbreviations: CAD = computer-aided detection, CEM = contrast-enhanced mammography, DWI = diffusion-weighted imaging, FA = flip angle, FOV = field of view, IR = inversion recovery, MRI = magnetic resonance imaging, NR = not reported, rCR = radiological complete response, RFOV = rectangular field of view, ROI = region of interest, TE = echo time, TR = repetition time

Supplemental S5:

Table S5 Patients with ypT0 and ypTis in meta-analysis and MRI enhancement of ypTis

Study	Total number of patients	ypT0 (n)	ypTis (n)	ypTis enhanced on MRI (n (%))	TP ypT0	TN ypT0	FP ypT0	FN ypT0	TP ypT0/is	TN ypT0/is	FP ypT0/is	FN ypT0/is
Bernardi (2022)[54]	51	16	12	8 (66.7)	30	16	0	5	22	20	8	1
Bodini (2004)[37]	73	3	4	3 (75.0)	60	1	2	10	57	2	5	9
Böttcher (2014)[38]	54	12	6	2 (33.3)	36	11	1	6	34	15	3	2
Chen (2008)[39]	51	22	6	1 (16.7)	15	21	1	14	14	26	2	9
De Los Santos (2011)[40]	81	23	9	6 (66.7)	51	13	10	7	45	16	16	4
Gampenrieder (2019)[41]	246	68	11	4 (36.4)	118	51	17	60	114	58	21	53
Hahn (2014)[42]	78	13	6	6 (100)	60	9	4	5	54	9	10	5
Hayashi (2013)[43]	264	66	32	25 (78.1)	175	36	30	23	150	43	55	16
Khazindar (2021)[45]	56	14	11	5 (45.5)	31	11	3	11	26	17	8	5
Lee (2017)[46]	17	4	2	0 (0)	9	3	1	4	9	5	1	2
Mirza (2016)[47]	69	11	6	2 (33.3)	47	8	3	11	45	12	5	7
Nakamura (2007)[48]	115	10	11	8 (72.7)	92	7	3	13	84	10	11	10
Negrao (2019)[49]	219	76	9	6 (66.7)	123	56	20	20	117	59	26	17
Santamaria (2019)[50]	82	19	8	6 (75.0)	55	11	8	8	49	13	14	6
Van Ramshorst (2017)[51]	297	112	69	16 (23.3)	76	97	15	109	60	150	31	56
Woodhams (2010)[52]	70	9	7	4 (57.1)	57	5	4	4	53	8	8	1
Zhang (2020)[53]	1031	309	60	41 (68.3)	657	146	163	65	616	165	204	46
Total	2854	787	269	143 (53.2)								
